# *Bacillus* Panophthalmitis with Posterior Extension to the Prechiasmatic Optic Nerve

**DOI:** 10.1155/2016/7652803

**Published:** 2016-11-22

**Authors:** James E. Kasenchak, Benjamin P. Hale, Thomas W. Wilson, Gregory M. Notz

**Affiliations:** Geisinger Eye Institute, 115 Woodbine Lane, Danville, PA 17822-5240, USA

## Abstract

A rare case of* Bacillus* panophthlamitis with extension to the prechiasmatic optic nerve secondary to hematogenous spreading after intravenous drug use is presented. A 27-year-old man with a recent history of trauma to the left eye presented with severe left eye pain following a binge of intravenous drug use. Visual acuity (VA) was LP. On examination he had chemosis, proptosis, elevated intraocular pressure, and a complete hyphema. CT-scan identified preseptal swelling, but no evidence of any posterior extension of the anterior process or orbital fractures. Topical and systemic therapy were initiated. On follow-up clinical examination less than 12 hours after presentation he had signs of a keratitis with worsening ophthalmoplegia and repeat imaging demonstrated posterior extension to the prechiasmatic optic nerve. Shortly after the cornea ruptured with cultures growing* Bacillus*. The patient underwent enucleation and has had no further progression of infection. To the best of our knowledge, this is the first report of* Bacillus* panophthalmitis presenting with signs of trauma with posterior extension to the prechiasmatic optic nerve.

## 1. Introduction

*Bacillus*-associated panophthlamitis has a fulminant course and early identification of the risk factors and potential presentations can assist in proper diagnosis and treatment. Risk factors include ocular trauma with a retained foreign body, postoperative complications, or hematogenous spreading. Presenting signs and symptoms may be similar to trauma, toxic anterior segment syndrome (TASS), angle closure glaucoma, carotid-cavernous fistula, or orbital cellulitis. In this paper, we describe a young man with a rapidly deteriorating course which required enucleation. Clinicians should be aware of this presentation and rapid clinical course to provide the most appropriate immediate treatment.

## 2. Case Presentation

A 27-year-old man presented to the emergency room with a 12-hour history of pain, proptosis, and decreased vision in his left eye. The patient has a past medical history of hepatitis C and is an active intravenous heroin abuser who admitted to using heavily the prior evening. Upon returning home he and his family stated that the eye looked normal. He slept for 12 hours and woke up with extreme left eye pain. He did report being hit in the area around 2 weeks prior but denied any more recent trauma to the eye.

On initial examination the visual acuity was 20/20 in the right eye and light perception in the involved left eye and tonometry was 38 mm Hg. The left eyelids were erythematous and edematous. The globe was proptotic with limited motility. There were extensive chemosis and trace subconjunctival hemorrhage. The cornea was diffusely edematous without an epithelial defect. The anterior chamber had a complete hyphema. The right eye examination was normal.

**Figure 1:**
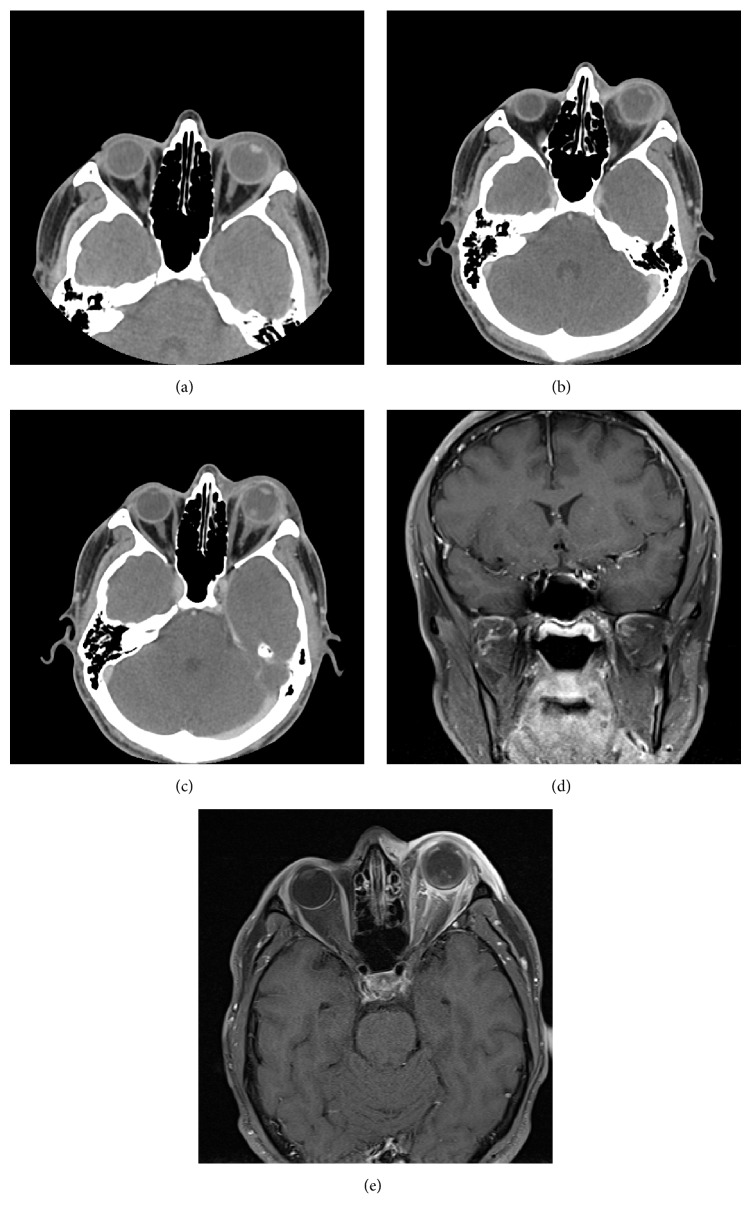
(a) Initial noncontrast CT with left periorbital soft tissue swelling. (b) Follow-up CT-scan with progressive postseptal extension of inflammation with shaggy appearance to the posterior sclera. (c) New vitreous opacity in the posterior globe. (d) MRI with thickening of the globe and scleral enhancement along the inner wall. The native intraocular lens has been displaced into the vitreous. (e) MRI with enhancement along the left optic nerve sheath to the prechiasmatic optic nerve.

The patient was afebrile and had no leukocytosis. HIV testing was negative. Initial CT orbits displayed periorbital soft tissue swelling without evidence of orbital fractures or sinus disease (Figure 1(a)). He received a dose of intravenous ampicillin and sulbactam and was discharged home on a course of topical prednisolone, atropine, dorzolamide, and timolol all twice a day. The following day the patient has no light perception in the left eye with a keratitis developing along with increasing pain, proptosis, and now almost complete ophthalmoplegia.

A repeat CT of the orbits identified retrobulbar fat stranding and thickening of the posterior sclera (Figure 1(b)). There was also now a focus of soft tissue density in the vitreous (Figure 1(c)). At this time consultation with a retina physician was obtained and the option of tap and inject or vitrectomy with intravitreal antibiotics was discussed. Tap and inject or vitrectomy was not performed as it was clear that this was a panophthalmitis with severe corneal melting and NLP vision.

Due to the keratitis and scleral involvement the patient was empirically started on intravenous vancomycin and started on fortified vancomycin and tobramycin ophthalmic drops every hour. MRI of the orbits, obtained less than 24 hours after initial presentation, demonstrated spontaneous dislocation of the native intraocular lens posteriorly into the vitreous with scleral enhancement along the inner walls of the left globe and enhancement was noted along the left optic nerve in the orbital apex extending through the optic canal to the prechiasmatic optic nerve (Figures 1(d) and 1(e)). Neurosurgery was asked to evaluate the patient and felt there was no indication for intracranial surgery at that time.

After 12 hours of intravenous and topical antibiotics a ring-shaped peripheral corneal abscess had developed. Due to the patient clinical worsening, cefepime was added with consultation from infectious disease. The following day the patient developed increased bloody drainage from the left eye. At this time the central cornea appeared ruptured. Cultures obtained grew* Bacillus*. Cefepime was discontinued and piperacillin/tazobactam was initiated. Enucleation was scheduled for the following day. Intraocular contents were cultured during the surgery and once again grew* Bacillus*.

A transesophageal echocardiogram was ordered and there was a small fibrous strand on the ventricular surface of the aortic valve, which was felt to possibly represent early stages of endocarditis. He remained afebrile but did develop a mild leukocytosis. His original blood cultures had no growth, but follow-up blood cultures did grow* Enterococcus faecalis*. He has had a difficult course since enucleation with multiple admissions for treatment of his infective endocarditis but each time has left against medical advice prior to completion of antibiotic therapy.

## 3. Discussion

*Bacillus* sp. were first recognized as a probable cause of panophthalmitis in 1890 with the first culture positive case reported in 1952 [1]. The initial cases were reported after penetrating trauma [2]. Endogenous panophthalmitis from* Bacillus*, although reported, is rare. [3–6]. It is thought that* Bacillus* sp. occur in patients who abuse injection drugs due to contamination of the drug mixture and injection paraphernalia. A transient bacteremia allows the* Bacillus* to gain access to ocular tissue [4]. Infections with* Bacillus* are commonly rapidly progressive even with aggressive treatment early. 70% of all eyes with culture positive* Bacillus* infection have progressed to losing their eye through enucleation/evisceration, even with the advent of intravitreal antibiotics [7].

Typical presentation of eyes infected with* Bacillus* described in exogenous and endogenous cases is poor visual acuity, chemosis, elevated intraocular pressure, pain, with rapid onset of proptosis, and a fulminant course [4, 8–10]. Previous reports of endogenous eye infections due to* Bacillus cereus* have also described a chocolate-brown exudate or blood stained hypopyon in the anterior chamber and a ring of edema adjacent to the limbus which develops on the cornea [2, 4, 10]. And as in our case, posterior subluxation of the lens has also been attributed to* Bacillus* endophthalmitis [9, 11].

Prior reports of* Bacillus* panophthalmitis have acknowledged the initial diagnosis to be incorrect, whether it be a thrombosis to the cavernous sinus [4] or angle closure glaucoma [9]. This is likely due to the rapid progression and poor history obtained from these patients who are in extreme pain and may be reluctant to provide an accurate history. In the absence of trauma, a recent history of intravenous drug use should alert the physician to signs of symptoms of panophthalmitis to consider* Bacillus* as a possible etiology. Furthermore, a hyphema in an IV drug user should arouse suspicion for a serious endogenous infection such as* Bacillus* or valvular endocarditis due to* Enterococcus faecalis* [12].

Our case once again underscores the need to be vigilant to find a rationale cause. Serial examination and imaging can often help with the diagnosis. Even without systemic signs or symptoms the ophthalmologist must be keenly aware of this fulminant infection and proper treatment guidelines. The history of trauma and presentation with pain, periorbital edema, and hyphema complicated the initial clinical decision making. The rapid clinical progression, well documented with CT and MRI imaging, with posterior extension to the prechiasmatic nerve is a unique and unusual finding among endogenous and exogenous* Bacillus* eye infections.

Generally, treatment of* Bacillus* endophthalmitis starts with vitrectomy with intravitreal amikacin/ceftazidime + vancomycin and dexamethasone at the time of surgery. Some authors suggest oral prednisone (1 mg/kg body weight) afterwards with an oral antibiotic such as ciprofloxacin [10]. It is not known whether this treatment protocol would have helped in this case as initial signs and symptoms were more of an orbital process that progressed to a panophthlamitis. The prognosis even with rapid vitrectomy remains poor [13].
